# Dissecting the Contribution of Release Factor Interactions to Amber Stop Codon Reassignment Efficiencies of the *Methanocaldococcus jannaschii* Orthogonal Pair

**DOI:** 10.3390/genes9110546

**Published:** 2018-11-12

**Authors:** David G. Schwark, Margaret A. Schmitt, John D. Fisk

**Affiliations:** Department of Chemistry, University of Colorado Denver, Campus Box 194, P.O. Box 173364, Denver, CO 80217-3364, USA; dschwark13@gmail.com (D.G.S.); margaret.schmitt@ucdenver.edu (M.A.S.)

**Keywords:** genetic code expansion, release factor 1, amber stop codon suppression, *M. jannaschii* orthogonal pair, fluorescence-based screen

## Abstract

Non-canonical amino acids (ncAAs) are finding increasing use in basic biochemical studies and biomedical applications. The efficiency of ncAA incorporation is highly variable, as a result of competing system composition and codon context effects. The relative quantitative contribution of the multiple factors affecting incorporation efficiency are largely unknown. This manuscript describes the use of green fluorescent protein (GFP) reporters to quantify the efficiency of amber codon reassignment using the *Methanocaldococcus jannaschii* orthogonal pair system, commonly employed for ncAA incorporation, and quantify the contribution of release factor 1 (RF1) to the overall efficiency of amino acid incorporation. The efficiencies of amber codon reassignments were quantified at eight positions in GFP and evaluated in multiple combinations. The quantitative contribution of RF1 competition to reassignment efficiency was evaluated through comparisons of amber codon suppression efficiencies in normal and genomically recoded *Escherichia coli* strains. Measured amber stop codon reassignment efficiencies for eight single stop codon GFP variants ranged from 51 to 117% in *E. coli* DH10B and 76 to 104% in the RF1 deleted *E. coli* C321.ΔA.exp. Evaluation of efficiency changes in specific sequence contexts in the presence and absence of RF1 suggested that RF1 specifically interacts with +4 Cs and that the RF1 interactions contributed approximately half of the observed sequence context-dependent variation in measured reassignment efficiency. Evaluation of multisite suppression efficiencies suggests that increasing demand for translation system components limits multisite incorporation in cells with competing RF1.

## 1. Introduction

To a first approximation, the process of translation involves the direct reading of three nucleotide codons in mRNA by three nucleotide anticodons of tRNAs employing standard Watson-Crick base-pairing rules. This simple picture of translation is incomplete. System composition, molecular modifications, and sequence context effects contribute to fine tune the efficiency of decoding individual codons in different mRNA contexts. System composition includes factors such as the relative concentrations of cognate and near cognate tRNAs, and in the case of non-sense suppression, suppressor tRNAs and release factors. The efficiency of competition is further modulated by tRNA modifications that impact tRNA–protein interactions as well as alter the energy of codon–anticodon pairings. The efficiency of decoding a particular codon within a particular mRNA sequence is additionally dependent upon the sequence context of the codon within the mRNA. Factors including tRNA–ribosome interactions, extended tRNA interactions with the mRNA (outside of the codon), and tRNA–tRNA interactions on the ribosome have been invoked as mechanisms to explain observed differences in decoding the same codon at different positions within an mRNA. A clear picture of the relative quantitative importance of these and other contributing factors to the efficiency of translation has not emerged (reviewed in [1,2,3]). To productively engineer translation better, quantitative understanding of the contributions of the multiple factors affecting the efficiency of translation is needed.

Proteins containing biosynthetically incorporated non-canonical amino acids (ncAAs) are finding increasing use in basic biochemical studies and biomedical applications. The majority of ncAAs are incorporated into proteins in response to an amber stop (UAG) codon using an orthogonal tRNA/aminoacyl tRNA synthetase (aaRS) pair. The tRNA is modified to include a CUA anticodon to fully Watson-Crick base pair with the amber stop codon. The aaRS is engineered to recognize and aminoacylate the ncAA onto the orthogonal tRNA. Variants of only two orthogonal tRNA/aaRS pairs, the tyrosyl tRNA/aaRS pair from *Methanocaldococcus jannaschii* and the pyrrolysyl tRNA/aaRS pair from *Methanosarcina* species have been engineered to incorporate the majority of ncAAs [4,5].

Despite the common ancestry of the orthogonal systems, the efficiency of ncAA incorporation is highly variable. Orthogonal tRNA/aaRS pairs evolved to recognize different ncAAs exhibit broadly different efficiencies. Individual orthogonal pairs also exhibit varying efficiencies depending on the site of the UAG codon in the target protein. Variability across pairs is related to the differing enzymatic efficiencies of evolved variants to recognize different ncAAs. Variability in the expression of components and the cellular availability of different ncAAs further contributes to observed differences in the efficiency of incorporation for different ncAAs. Variability in the efficiency of incorporation based on the placement of a target codon within a gene sequence is the result of multiple differential competing interactions of tRNAs and release factors with the mRNA and the ribosome. The relative quantitative contributions of the multiple factors that determine the overall efficiency of incorporation of amino acids in response to amber stop codons are largely unknown.

Genetic code expansion via amber stop codon reassignment has been limited to incorporation of ncAAs at a single site because of competition with termination signals that curtail the amount of protein produced when multiple suppressions are attempted in a single protein. Recently, the Sakamoto, Wang, and Church laboratories each engineered genomic changes in *Escherichia coli* that mitigate the usual cytotoxic effect of deletion of the release factor that competes for decoding the amber stop signal [6,7,8]. The efficiency of incorporation of amino acids in response to amber stop codons is improved in these strains relative to strains that express release factor 1 (RF1); however, the contribution of release factor competition to the overall efficiency of amber stop codon reassignment has not been quantified.

To better understand the factors that contribute to the efficiency of reassigning amber stop codons in *E. coli* using the *M. jannaschii* tyrosyl tRNA/aaRS pair, the efficiency of suppression of individual and combinations of amber stop codons in green fluorescent protein (GFP) were quantified. To a first approximation, the system composition and tRNA modifications in each of these cases are expected to be identical. As a result, the suite of interactions directed by the mRNA sequence surrounding the targeted amber codon is expected to be a primary contributor to observed differences in the efficiency of stop codon suppression. The contribution of competition between the suppressing tRNA and release factors to amber codon reassignment efficiency was evaluated by quantifying the fluorescence of the GFP reporter variants in related *E. coli* strains with and without the competing release factor RF1. Evaluation of efficiency measurements of multiple reassignments within a single GFP gene enabled quantification of the contribution of competition and indicated that orthogonal pair machinery becomes a limiting factor during over-expression of genes containing multiple amber stop codons.

Systematic experimental manipulations of the translation system composition directed at quantitative evaluation of translational efficiency have not been reported. Manipulations with the purpose of improving protein production of mammalian genes in bacterial systems have qualitatively indicated that low abundance tRNAs can limit translational efficiency [9]. In the context of ncAA incorporation, multiple vector systems have been developed to improve the efficiency of ncAA incorporation through changes in the expression of orthogonal components, but systematic analyses of compositional changes between vector systems have not been performed [10,11,12]. Cell size, ribosome and tRNA numbers, and the ratio of ribosomes to tRNAs all vary with growth rate, making comparisons between experiments difficult. Experiments that report on codon-specific translational efficiencies have only recently become available (e.g., single molecule and ribosome profiling). The quantitative contribution of system composition variability to translational efficiency is largely unknown.

Beyond variability due to system composition, the sequence context in which a nonsense codon appears also modulates the efficiency of natural nonsense suppression [13,14]. Direct measurement of sequence context effects in sense codon reading are also evident from single molecule experiments and ribosome profiling, but systems that provide simple read-outs for sense codon reading are not available [2]. Related to context-specific reading differences, synonymous codons read by the same tRNA are decoded at different rates [15]. Both the efficiency of programed reading frame shifts and gain of function missense mutations are sequence context-dependent [16,17,18]. Evaluating the quantitative contributions of system composition and sequence context effects on the efficiency of nonsense suppression offers a window into the details of translation.

Context effects are of particular importance for genetic code expansion studies as the choice of substitution position within a target protein can have a large effect on the efficiency of ncAA introduction. In the case of amber suppressor tRNAs, the identity of the nucleotides immediately upstream and downstream of the amber codon in the mRNA have been shown to affect reading efficiency [19,20]. The bases in the vicinity of the amber stop codon are numbered such that the amber stop codon positions are +1, +2, +3, the 5′-nucleotides are assigned negative numbers and the 3′-nucleotides are assigned positive numbers. The magnitude and direction of context effects on amber stop codon reassignment with natural suppressor tRNAs depends on the specific suppressor tRNA employed. That context effects depend on the framework of the reassigning tRNA suggests that interactions outside of the codon-anticodon region contribute to observed modulations of reading efficiency. Evidence exists both for and against extended interactions of a tRNA with the mRNA sequence [19,20,21], indirect effects of tRNA-ribosome interactions [22,23], and termination factor-mRNA interactions [3,24] contributing to the context-depending efficiency of stop codon reassignment.

Data on multiple natural and engineered amber stop codon suppressors inserting different amino acids into the same seven test positions in a Lac repressor protein showed efficiencies spread over an approximately 10-fold range [25,26,27,28]. Different suppressor tRNAs showed different degrees of context effects and exhibited different patterns of relative increases and decreases in efficiency at the different positions [25,26]. Preferred codon contexts are evident through sequence analysis; however, the function of preferred contexts is not clear [29,30,31,32,33,34]. Although some codon orderings are not found in protein coding genes and different patterns of codon usage appear in highly and weakly expressed genes, simple rules regarding sequence preferences around individual codons are not evident [30,35]. Identifying signatures of codon context bias through bioinformatics analysis is confounded by the necessity of correcting for codon usage, tRNA abundances, GC content, and amino acid frequencies in proteins. Furthermore, the context of codons in naturally selected genes could be the result of evolutionary pressure to either increase or decrease codon reading efficiencies, resulting in confusion based upon examination of sequence alone [30,31,36,37].

Three recent works have presented data that can be analyzed to shed light on the context-specific reading of amber codons by the *M. jannaschii* and *Methanosarcina mazei* orthogonal tRNA/aaRS systems most commonly used in genetic code expansion [12,38,39]. Two of these studies were directed at generating improved systems for ncAA incorporation; however, the data presented can also be used to evaluate the effect of codon context on the efficiency of translation. The third study specifically addressed sequence context effects on the efficiency of amino acid incorporation by the *M. mazei* orthogonal tRNA/aaRS pair.

In a study describing the development of an improved vector system for the expression of ncAA-incorporating orthogonal tRNA/aaRS pairs, Young et al. measured the incorporation of 15 non-canonical amino acids at three amber stop codon positions in GFP [12]. The three sequence contexts in which suppression of the amber stop codon was evaluated showed an approximate 10-fold range of efficiencies, similar to that observed for natural amber suppressor tRNAs [25,40]. The ncAA incorporation efficiencies varied both with the particular evolved *M. jannaschii* aaRS used (and therefore, the identity of the amino acid) and with the position of the amber stop codon in the GFP reporter protein. Different patterns of relative efficiencies at the three sites were observed for different evolved aaRSs.

Pott et al. [39] selected optimized sequence contexts for the introduction of amino acids in response to an amber stop codon in an appended tag. Selection experiments were performed on libraries of N-terminal tag sequences containing an amber stop codon between two randomized codons (NNN-NNN-UAG-NNN-NNN). The nucleotide sequence contexts selected for improved ncAA incorporation using the *M. jannaschii* and *M. mazei* systems were similar, but not identical. The selected sequences increased the efficiency of amber stop codon suppression three-fold to five-fold relative to randomly chosen sequences. The preferred context for the *M. jannaschii* pair included a strong preference for adenosine (A) in the position immediately following the UAG stop codon, as had been previously observed for a number of natural amber stop codon suppression systems [24,25]. Additional strong preferences at the −5, −2, −1, +6 and +7 positions were also identified. Interestingly, the sequence contexts selected for efficient tyrosine and lysine sense codon reading were decidedly different than the contexts selected for amber stop codons. The different contexts for sense and nonsense codons suggests selection against release factor-mRNA interactions in the amber stop codon cases. The utility of the data presented for evaluating context effects on the orthogonal tRNA/aaRS systems was limited by the small number of selected sequences (fewer than 10 per selection) and an incomplete characterization of the efficiency of amber stop codon suppression for the selected sequences [39].

Codon context effects were explicitly examined for the *M. mazei* pyrrolysyl tRNA/aaRS orthogonal pair by Xu et al. employing a β-galactosidase assay to evaluate suppression efficiencies of a library containing randomized codons upstream and downstream of the targeted UAG codon (NNN-UAG-NNN) [38]. The overall consensus sequence for strong amber stop codon suppression identified was contained within the consensus sequence selected by the Pott et al. evaluation. The Pott et al. evaluation identified additional, stronger sequence effects based on the identity of the nucleotides at positions −6, −5, +7, +8 and +9, sites not analyzed by Xu et al. In the Xu et al. study [38], the efficiency of suppression across the evaluated sequence contexts varied over a 100-fold range, much larger than had been previously observed for natural suppressors. The relative importance of specific bases was difficult to interpret as many of the single base mutants were not reported. In cases where comparable single and double mutants were evaluated, the effects of the mutations on amber stop codon suppression efficiency appeared to be largely non-additive. Evaluation of the set of 18 single site mutants away from the consensus sequence indicated that the efficiency of suppression was exquisitely sensitive to the exact sequence.

Unraveling the mechanistic sources and quantitative contributions of the many factors affecting the overall efficiency of translation of a given codon is a largely open question and represents an important next step in quantitatively understanding the process of translation. The generation of high-quality quantitative efficiency measurements on a very large set of related systems in which individual variables are modulated would provide a necessary data set to begin to decipher base-level contributions to translational efficiency.

## 2. Materials and Methods

### 2.1. Cell Strains

DH10B: F^−^ mcrA Δ(*mrr-hsd*RMS*-mcr*BC) Φ80d*lac*ZΔM15 Δ*lac*X74 *end*A1 *rec*A1 *deo*R Δ(*ara,leu*)7697 *ara*D139 *gal*U *gal*K *nup*G *rps*L λ^−^ (Thermo Fisher, Waltham, MA, USA).

C321.ΔA.exp: *E. coli* MG1655 Δ(ybhB-bioAB)::zeoR ΔprfA; all 321 UAG codons changed to UAA.

C321.ΔA.exp was a gift from George Church (Addgene reference # 49018, Watertown, MA, USA) [41].

### 2.2. General Reagents and Materials

All restriction enzymes and DNA polymerases were purchased from New England Biolabs (Ipswich, MA, USA) and used according to the manufacturer’s instructions. ATP was purchased from Fisher (BP413-25) (Waltham, MA, USA) and dNTPs were purchased form New England Biolabs (N0447S) (Ipswich, MA, USA). DNA isolation was performed using a Thermo Scientific GeneJET plasmid miniprep kit (K0503) (Thermo Scientific, Waltham, MA, USA) according to the manufacturer’s protocols.

Luria broth (LB) liquid media (per liter: 10 g tryptone, 5 g yeast extract, 5 g NaCl) and LB agar plates with 15 g/L agar (TEKNova, A7777, Hollister, CA, USA) were used unless otherwise noted. Isopropyl-β-d-thiogalactoside (IPTG) was purchased from Gold Bio (I2481C5) (St. Louis, MO, USA). Spectinomycin (Enzo Life Science, BML-A281, Farmingdale, NY, USA) was used at 50 μg/mL to maintain the pUltra-based vectors harboring the tRNA and aaRS genes. Carbenicillin (Thermo Scientific, Waltham, MA, USA) was used at 50 μg/mL to maintain the vectors harboring the GFP reporter gene. All bacterial cultures were grown at 37 °C unless otherwise noted.

Electrocompetent stocks of all strains were prepared in-house according to the method of Sambrook and Russell [42]. Typical transformation efficiencies for electrocompetent cells produced in this way are 10^9^ cfu/μg of supercoiled DNA. All transformations were recovered in SOC (20 g/L tryptone, 5 g/L yeast extract, 10 mM NaCl, 2.5 mM KCl, 10 mM MgCl_2_, 20 mM glucose) for 1 h at 37 °C with shaking prior to transfer to media containing the appropriate antibiotics.

All oligonucleotides were purchased from Integrated DNA Technologies (Coralville, IA, USA). All DNA sequencing was performed by Genewiz (Plainfield, NJ, USA).

### 2.3. Construction of Amber Stop Codon-Containing GFP Reporter Variants

The complete sequence of the 100% fluorescent, wild-type GFP reporter plasmid has been reported (Supporting information in [43]). Amber stop codon containing variants were based on this vector.

Mutagenesis for the preparation of the GFP variants was achieved by using a protocol modified from the Stratagene QuikChange Multi-Site Directed Mutagenesis kit (San Diego, CA, USA). Briefly, 125 ng of mutagenic primer (one or two primers used per site mutated), 100 ng of template plasmid DNA, Q5 High Fidelity Polymerase Buffer (1X final concentration), dNTPs (200 µM final concentration), and Q5 DNA polymerase (0.5 units) were combined into a 200 µL PCR tube. Ultrapure water was added to adjust the final reaction volume to 25 µL. Reactions were then subjected to thermocycling in either a PeqStar (peqLab, Wilmington, DE, USA) or MjMini (Bio-Rad, Hercules, CA, USA) thermocycler. After initial denaturing at 98 °C for 1 min, 25 cycles of the following were performed: 98 °C for 20 s, 72 °C for 20 s, and 72 °C for 15 s/kb of template plasmid. After 25 cycles were completed, a final 5 min extension at 72 °C was performed, and the reactions were then cooled down to 4 °C. Following thermocycling, 1 µL (20 units) of DpnI restriction enzyme was added to the reaction tube and incubated for 2 h at 37 °C. Reactions were transformed into electrocompetent *E. coli* DH10B without cleanup.

The sequences of each mutagenic primer are provided in Appendix A, Table A1.

### 2.4. Vector for Expression of the Orthogonal *M. jannaschii* tRNA and aaRS

The vector containing the genes for expression of the *M. jannaschii* tyrosyl tRNA and aminoacyl tRNA synthetase has been described [44]. Expression of the aaRS is driven by a constitutive lpp promoter in the system used in this evaluation.

### 2.5. Fluorescence-Based Screen for Quantification of the Efficiency of Translation

The fluorescence-based screen provides a quantitative measure of the extent of amber stop codon suppression at each test position by bracketing the observed GFP signal for codon reassignment measurement between a “100% fluorescence” reference value produced by expressing superfolder GFP with a tyrosine UAC codon specifying the fluorophore and a “0% fluorescence” reference value established through expressing superfolder GFP with a non-tyrosine codon specifying the fluorophore. Both the 100% and 0% fluorescence reference systems include a plasmid expressing the *M. jannaschii* aaRS and tRNA to maintain a metabolic burden on the cells equivalent to that of the systems under evaluation.

The fluorescence-based screen for sense codon reassignment has been described [43,44]. Briefly, a given GFP reporter vector was co-transformed with the vector expressing the orthogonal translational components. After overnight growth, colonies were picked into 200 μL LB media in a 96-well plate. Cells were grown to at least mid-log phase (usually 8–10 h) with shaking at 37 °C. Cells were diluted 10-fold into media containing 1 mM IPTG to induce expression of the GFP reporter. Assays were performed in a Fluorotrac 200 clear bottom 96-well plate (Greiner 655096, Kremsmünster, Austria) and monitored in a BioTek Synergy H1 plate reader (BioTek, Winooski, VT, USA) at 37 °C with continuous double orbital shaking. The optical density (OD_600_) and fluorescence of each well was measured every 15 min for at least 15 h; optical density was measured at 600 nm, and fluorescence was measured with an excitation at 485 nm and detection at 515 nm with an 8 nm band pass.

Calculation of the efficiency of codon reassignment has been described in detail in supplementary section S6 of Schmitt et al. [43]. Briefly, the relative fluorescence of each 200 µL culture was calculated by dividing the 0% reassignment control-blanked fluorescence for a reporter variant by the path length and blank corrected OD_600_ at each time point. The 100% relative fluorescence unit (reported as fluorescence units per unit optical density at 600 nm, RFU) value for amber codon reassignment efficiency was defined by taking an average of six separate colonies expressing wild type GFP and the orthogonal machinery. For each sample well, the relative fluorescence was calculated for each of the data points gathered between 8 and 12 h after induction of GFP with IPTG. These relative fluorescence values were averaged to determine the overall RFU for each sample well. A more detailed explanation of time point selection is provided in section S7 of Schmitt et al. Amber codon suppression efficiency for each UAG positional variant was calculated by dividing the overall RFU values from a single clone by the average 100% reference RFU value [43].

For a given GFP reporter vector, the reported amber codon reassignment efficiency is the average of at least six biological replicates. The majority of suppression efficiencies reported here are the average of between 12 and 18 biological replicates. The calculated efficiencies for only two reporter vectors, the two amber stop codon containing variants Y143/Y182 and Y106/Y143, in the C321.ΔA.exp cells are the average of fewer than six biological replicates (four and three, respectively).

## 3. Results and Discussion

In order to better understand the factors controlling the overall efficiency of amber stop codon suppression by the orthogonal *M. jannaschii* tyrosyl tRNA/aaRS pair, we employed a fluorescence-based screen to quantify amber stop codon reassignment efficiencies at eight different tyrosine positions in superfolder green fluorescent protein in two *E. coli* strains. To remove complicating effects resulting from differences in protein folding and performance between mutants and control proteins, the suppression of amber stop codons was accomplished with the tyrosine-incorporating orthogonal *M. jannaschii* tRNA/aaRS pair, and stop codons were placed at tyrosine positions in each GFP reporter variant. In this way, the protein produced by amber stop codon suppression with the *M. jannaschii* pair is phenotypically identical to wild type GFP controls, allowing fluorescence to be a direct measurement of amber stop codon reassignment efficiency. The mRNA sequence surrounding each amber stop codon was the only difference between the eight single amber codon GFP reporters. The identity and the expression levels of the orthogonal *M. jannaschii* tyrosyl tRNA and aaRS, the promoter driving the GFP reporter gene, and the cell lines used (i.e., system composition/cellular environment) were all consistent between the evaluated systems. Under these conditions, differences in decoding efficiency observed between amber stop codon positions should be predominantly due to codon context effects. The amber stop codon suppression efficiencies were evaluated in two *E. coli* strains, with and without the competing release factor RF1.

The hypothesis that the efficiency of suppression of multiple amber stop codons within a single gene is independent and additive was then evaluated for a series of related GFP variants. The measured amber stop codon suppression efficiencies for the single amber stop codon variants were used to predict the suppression efficiencies of GFP variants containing multiple amber stop codons, and the predicted and measured amber stop codon reassignment efficiencies for 14 multiple amber stop codon-containing GFP variants were compared. Comparison of reassignment efficiencies between the two strains enabled the isolation of the contribution of release factor interactions to the overall efficiency of translation.

### 3.1. Measurement of Reassignment Efficiencies for single UAG GFP Variants using the *M. jannaschii* Tyrosyl tRNA/aaRS Pair

In order for GFP fluorescence to accurately quantify successful incorporation of an amino acid in response to a stop codon, translation of the reporter variants in the absence of the amber stop codon reassignment machinery must result in non-fluorescent protein products. Singly, each of the nine tyrosine codons within the wild type GFP reporter gene (which served as the 100% fluorescent control) were mutated to the amber stop codon. GFP fluorescence was monitored in the absence of orthogonal translational machinery for each variant. No significant fluorescence from the GFP products for eight of the nine single amber stop codon variants was observed. One variant, Tyr 237 UAG, showed GFP fluorescence above that of the 100% reference wild type GFP. The observation of wild type levels of functional GFP in this system is unsurprising. Position 237 is the second to last residue in the GFP sequence, and stopping translation of the reporter variant so close to the C-terminus would not be expected to dramatically decrease function. The Tyr 237 UAG mutant was not utilized further.

Amber stop codon reassignment efficiency was measured with the *M. jannaschii* tyrosyl tRNA/aaRS pair for each of the eight GFP variants in *E. coli* DH10B cells. The measured incorporation efficiencies, reported as the cell density corrected fluorescence of the stop codon suppressing system divided by the cell density corrected fluorescence of a system in which wild type GFP was expressed, ranged from 51.2 ± 1.9% to 117.0 ± 2.6% (Table 1). The suppression efficiency reports on a combination of enzyme efficiency and relative expression level for the particular orthogonal system employed. The average reassignment efficiency across the eight positions measured is 87%, a value on the high end of the efficiencies measured for natural suppressors. The context-dependent variation of approximately two-fold observed for the *M. jannaschii* pair in this experimental evaluation is in line with that observed for natural suppressors. Typically, the efficiencies of more effective suppressors are less dependent on sequence context.

The identity of the nucleotide on the 3′-side of the UAG codon (i.e., +4 position) was the first recognized sequence context effect [28]. Multiple mechanistic rationales for the influence of a 3′-base on translational efficiency have been put forth, but the actual mechanisms remain unclear. An A or G immediately 3′ of the amber codon could potentially base pair with the universally conserved U33 of tRNAs. An alternative hypothesis for the preference of purines at the +4 position is that base stacking stabilizes the suppressor tRNA–mRNA interactions. Changing the nucleotide following the amber stop codon from C to A resulted in a 10-fold increase in the efficiency of suppression for the SupE amber suppressor [27,28]. The trend of purines rather than pyrimidines in the +4 position leading to improved translation efficiency was generally supported in a large study employing multiple suppressing tRNAs [27]. Context effects differed in direction and magnitude for the different suppressors in the same mRNA context. That context effects are different for different suppressor tRNAs suggests that the nucleotide in the +4 position of the mRNA is not the only factor involved in determining the efficiency of translation of a given codon [19,20]. An additional hypothesis, at least for the case of amber stop codon suppression, is that differential competition between interactions with release factors contributes to context effects [24].

The data gathered in our investigation generally support the hypothesis of +4 purines improving the efficiency of incorporation in response to an amber stop codon. The sequence contexts of the UAG codons evaluated include cases where the −1 bases are U, C and A and the +4 bases are C, A and G (Table 1). Three of the four most efficient amber suppressions with the orthogonal *M. jannaschii* tRNA/aaRS were observed in a context with a +4 A. In contrast, three of the four least efficient amber stop codon suppressions were in the context of a +4 C. A trend for the effect of the identity of the nucleotide at the −1 position on the efficiency of translation was not observed. This finding is in line with previous studies that did not identify clear patterns of context effects as a result of the nucleotides upstream of the amber stop codon (i.e., positions −1, −2, −3) [40].

### 3.2. Role of Ribosomal Release Factor 1 in Determining Amber Stop Codon Reassignment Efficiency

**Figure 1:**
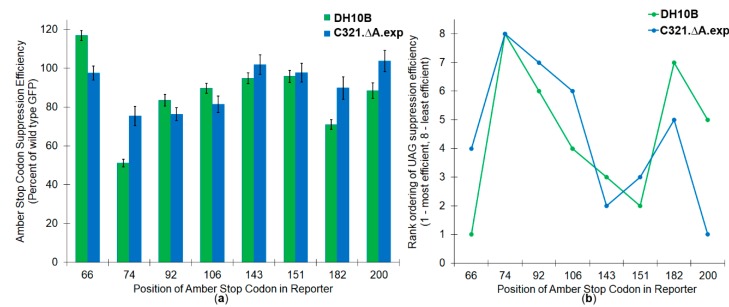
Side by side comparison of amber stop codon reassignment in two *Eschericia coli* strains. (**a**) Reassignment efficiencies as a percentage of wild type GFP at each of the eight single site UAG variants. (**b**) Rank ordering of suppression efficiencies of eight single site UAG positions from most efficient (placed at #1) to least efficient (placed at #8) in two *E. coli* strains. Green bars and dots represent data measured in DH10B cells; blue bars and dots represent data measured in C321.ΔA.exp.

One of the predominant hypotheses regarding the source of context effects posits that differential interactions between the protein release factor and the mRNA contribute to observed sequence-dependent differences in the efficiency of amber suppression. Within the literature, studies both support and refute the role of release factor interactions in governing context effects [1,28]. In order to evaluate the contribution of release factor mRNA interactions in determining amber stop codon suppression efficiencies by the orthogonal *M. jannaschii* tRNA/aaRS pair, the set of amber stop codon GFP mutants were further evaluated in a genomically recoded *E. coli* strain, C321.ΔA.exp, in which all instances of the UAG codon in the genome were replaced, and the gene coding for ribosomal RF1 was removed [41] (Figure 1, Table 1). The single amber stop codon GFP variants were suppressed with efficiencies between 75.5 ± 4.9% and 103.7 ± 5.5% in the genomically recoded *E. coli* strain, compared to 51.2 ± 1.9% to 117.0 ± 2.6% in *E. coli* DH10Bs.

Utilization of near-cognate tRNAs to resolve issues of the ribosome stalling at the UAG codon in RF1 deleted cell lines has been observed [45]. In order to evaluate whether incorporation of non-tyrosine amino acids in response to amber codons could be contributing to the observed fluorescence when the orthogonal translation machinery and GFP reporters were expressed in C321.ΔA.exp, we examined two of the single site GFP reporters (positions 66 and 74) in concert with an inactive *M. jannaschii* tRNA variant. In these systems, the metabolic burden placed on the cells is as similar as possible to the systems under which codon reassignment is evaluated. In the position 74 case, any observable fluorescence above the intrinsic fluorescence of the medium could be indicative of background suppression. This statement assumes that position 74 in GFP is tolerant to mutation and able to fold and fluoresce with a different amino acid substituted for tyrosine. In the case of the position 66 mutant, only background incorporation of tyrosine would produce fluorescence. Significant fluorescence above background media, particularly within the time window over which reassignment efficiency is quantified, is not observed in these systems. This result suggests that, at least for the combination of orthogonal translation machinery, GFP reporters, and the cellular environment (e.g., media, temperature, antibiotic concentration) utilized in this study, suppression of amber codons by endogenous, near-cognate, non-tyrosine tRNAs is not a major contributor to the fluorescence observed in the RF1-deleted cell line. Further, an overestimation of the possible suppression by near-cognate tRNAs would contribute at most 0.006% to the calculated reassignment efficiency. A more elaborate discussion of these points is included in Appendix B.

The general trend of the relative efficiency of stop codon reassignment at the various positions was similar between the two cell lines. Figure 1b shows the rank ordering of the position of the most efficiently suppressed amber stop codon (1) to the position of the least efficiently suppressed amber stop codon (8) in each strain. While rank ordering was similar, there were significant differences in observed amber stop codon suppression efficiencies between the two cell lines. Three of the four least efficiently suppressed amber stop codon positions in *E. coli* DH10B cells (74, 182, and 200) were all significantly improved in the RF1 deleted cell line (+24.3, +18.8, and +15.2%, respectively). Three of the positions tested (66, 92, 106) showed decreases in absolute amber stop codon suppression efficiency upon removal of RF1 (−19.4, −7.2, and −8.2%, respectively).

A measure of the extent of the contribution of RF1 to the observed context effect can be derived from the deviation in measured values. The differences between measured stop codon suppression efficiencies in the two strains were statistically significant for seven of the eight single site variants evaluated (*p*-values less than 0.001, Table 1). These differences suggest that interactions with RF1 contribute to the variation in site to site reassignment efficiencies at all positions evaluated except position 151. The larger spread of measured efficiencies in the cells expressing RF1 (DH10B) suggests that both tRNA and release factor interactions are contributing to context effects. The extent of the context effect in DH10B is approximately 20% of the average value (i.e., the standard deviation of the average of reassignment efficiencies at the 8 positions). In contrast, the context effect in C321.ΔA.exp cells is approximately 11% of the average value. The difference in context effects between the two systems suggests that the contribution of release factor interactions accounts for about half of the observed context effect in DH10B cells.

The +4 sequence contexts for the set of codon positions that showed increased amber stop codon reassignment efficiencies upon removal of RF1 were consistent, having a +4 C and either U or C at the −1 position relative to the amber stop codon. Sequence consensus was not apparent across the positions that showed reduced or unchanged amber stop codon suppression efficiency upon RF1 removal. The consistency of the sequence contexts and the direction of changes in suppression efficiency suggest that RF1 does play a role in determining context effects. Our data suggests that RF1 has a preference for contexts with a +4 C.

### 3.3. Measurements of Amber Stop Codon Reassignment Efficiencies for Multiple UAG-Containing GFP Variants

The synergy/independence of the suppression of multiple amber stop codons in a single gene has not been subjected to widespread systematic analysis and could be useful in evaluating the contribution of local and global effects on context-dependent variations in codon translation. Recent manuscripts have evaluated the overall efficiency of ncAA incorporation in response to multiple amber codons in single genes in the C321.ΔA.exp cell line, but these data have not been evaluated in relation to deciphering the factors effecting amber codon reassignment efficiency [45,46]. The proposed mechanisms for mRNA sequence context effects on translation efficiency predominantly invoke local interactions and suggest that the effects of context on multiple incorporation events within a single gene would be additive and independent. To be clear, “additive and independent” interactions would imply that if, for example, two different amber stop codons are individually suppressed with 50% efficiency, then a double mutant containing the combination of the two amber stop codons would be expected to be produced at 25% overall efficiency. The measured single site suppression efficiencies were used to predict multisite suppression efficiencies based on an assumption of additivity and independence (Table 2).

**Figure 2:**
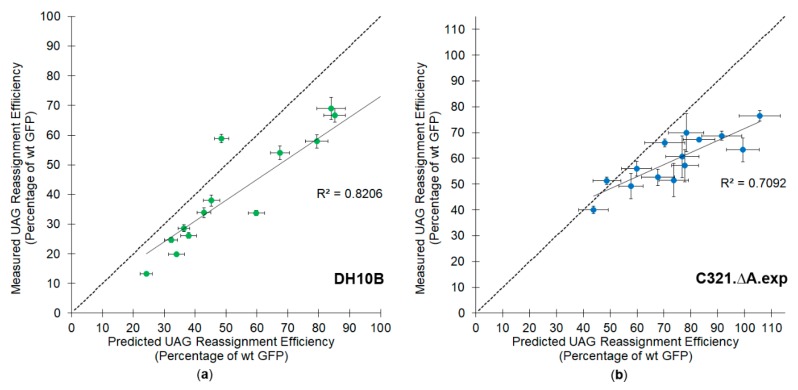
Measured versus predicted amber stop codon reassignment efficiencies for GFP reporter vectors containing two or more suppressible amber codons in (**a**) *E. coli* DH10B; (**b**) *E. coli* C321.ΔA.exp. The dotted line on the diagonal represents where the data would be expected to fall based on an assumption of additivity and independence for the efficiency of multisite amber stop codon reassignment. The full line represents the linear regression analysis of the data, with the *R*^2^ value shown on each graph.

The amber stop codon suppression efficiencies for 14 different double, triple, quadruple and quintuple tyrosine to amber stop codon GFP mutants were measured in both *E. coli* DH10B and C321.ΔA.exp cells (Table 2). As expected, the overall UAG suppression efficiencies decrease with increasing numbers of UAG codons in the GFP reporter. In both cell lines there is a clear trend that the measured amber stop codon suppression efficiencies are consistently less than what would be predicted from the measured single site efficiencies (Figure 2). In the DH10B cell line, for all but one of the variants, the measured amber stop codon reassignment efficiencies were lower than the predicted efficiencies. In the RF1 deleted cell line, the measured amber stop codon reassignment efficiencies were consistently lower than the efficiencies predicted for non-interacting independent sites, but the measurements were more varied, reflecting greater variability in the growth of the C321.ΔA.exp cells. We analyzed the difference between the predicted and measured distributions with a two-factor analysis of variance (ANOVA) statistic. The measured efficiencies differ from those predicted using the assumption of additivity with *p*-values of 0.00054 (DH10B) and 0.00025 (C321.ΔA.exp). The observed trends suggest that some factor contributing to the efficiency of translation is exerting a synergistic as opposed to independent effect.

The most probable explanation for synergy involves variation in relative translational potential as a function of demand. As demand for orthogonal machinery (e.g., aminoacylated tRNA) increases with the number of suppressible amber codons in an mRNA, the reassignment efficiency at each individual site might be expected to decrease. Additional sources of synergistic interactions might involve differential ribosome drop off frequencies or mRNA degradation times related to overall translation efficiency [47,48].

To evaluate the contribution of competition between the available orthogonal tRNA and the release factor to the efficiency of amber codon reassignment, the average per site reassignment efficiencies were calculated for cases of increasing demand (1 vs. 2 vs. 3 amber codons per gene). The comparison between the average per site reassignment efficiency for the strains with and without release factor suggests that competition is occurring in the RF1-containing strain (DH10B), but is largely absent from the RF1 deleted strain (Table 3). The average per site reassignment efficiency drops from 87% for single reassignments per gene to 67% for genes requiring five reassignments in the RF1 containing cell line. In the RF1 deleted cell line, C321.ΔA.exp, the calculated per site reassignment efficiency is fairly constant at 85% for genes containing between one and five suppressible amber codons.

Perhaps unexpectedly, the average per site incorporation efficiency appears to plateau at approximately 67% for reporters with four and five UAG codons, rather than decreasing as was observed going from reporters with 1 UAG codon to 2 UAG codons. Our tentative explanation of this plateau is that an equilibrium is being reached between the combined kinetics of incorporation and tRNA recharging for the particular concentrations of *M. jannaschii* tRNA, *M. jannaschii* aaRS, RF1, and available tyrosine present in the cell. At lower codon demand (e.g., fewer suppressible stop codons per mRNA), the concentrations of aminoacylated tRNA and release factor in the cell favor orthogonal pair directed amber codon reassignment. As the number of suppressible stop codons per mRNA increases, the functional steady-state level of aminoacylated tRNA decreases, leading to lower per site reassignment efficiency. The drop-off is not expected to be linear; increased concentrations of non-aminoacylated tRNA increases the rate of re-aminoacylation. We expect that at some point a kinetic equilibrium is established with a particular, system-dependent per site efficiency. This explanation accounts for the little to no decrease in per site efficiency observed in C321.ΔA.exp as the number of UAG codons in each reporter vector increased. In these cells, aminoacylated tRNAs are not competing with RF1 for the amber stop codon. The available tRNAs are able to be aminoacylated, participate in translation, and be recharged for participation in another peptide bond-forming reaction without being kinetically outcompeted by RF1 during that course of events.

### 3.4. Effect of Altering the Genetic Code on the Health of Amber Stop Codon Reassigning Systems

The extent to which cells tolerate reassignment of a stop codon to an amino acid has not been widely investigated. Missense errors are thought to be generally destabilizing, but translation errors introduced through defective aaRS editing or antibiotic treatment are broadly tolerated by cells. We examine the extent to which reassignment of the amber stop codon is tolerated by *E. coli* using the measured instantaneous doubling times of each reassignment system (Table 4). The requirement for cells to grow and divide in the presence of two antibiotics while replicating the DNA for both the orthogonal translation machinery vector and a GFP reporter vector slows the growth of both DH10B and C321.ΔA.exp. Allowing amber stop codon suppression by expressing the active *M. jannaschii* tRNA/aaRS pair exerts an additional deleterious effect on DH10B cells. Slowing of the instantaneous doubling times of C321.ΔA.exp beyond that which comes as a result of the antibiotic presence is not observed. Although C321.ΔA.exp cells exhibit longer doubling times than *E. coli* without the RF1-related genomic changes, amber codon reassignment does not have as much of an effect on the health of C321.ΔA.exp relative to the effect amber codon reassignment exerts on the health of DH10B.

## 4. Conclusions

We present a high-quality data set of amber stop codon suppression efficiency measurements employing a GFP fluorescence-based screen to quantify amber suppression by the orthogonal *M. jannaschii* tyrosyl tRNA/aaRS pair. Comparisons between sites of amber codon suppression within genes and between cells with and without competing release factor allow the quantification of some system composition-dependent contributions to orthogonal pair-directed nonsense suppression efficiency. Because transition is an incredibly complex process, no single set of measurements can define in an absolute way the contribution of particular factors to the overall process. The quantified values described here are a small step toward building a much larger set of quantitative measurements needed to describe the ways in which various system composition variables contribute to the overall activity of the translational apparatus. The data presented should help inform the use of the *M. jannaschii* pair in genetic code expansion applications. Measurements made using orthogonal machinery to incorporate natural amino acids are a useful tool for dissecting the workings of the natural translational apparatus.

Amber stop codon reassignment efficiencies for eight single stop codon GFP variants ranged from 51.2 ± 1.9 to 117.0 ± 2.6% in *E. coli* DH10B and 75.5 ± 4.9% to 103.7 ± 5.5% in the RF1 deleted *E. coli* C321.ΔA.exp. The relative ordering of reassignment efficiencies in the two strains was similar, suggesting that RF1 contributes to but is not a dominant factor for the observed context-dependent variation in translational efficiencies. Evaluation of reassignment efficiency changes in specific sequence contexts in the presence and absence of RF1 suggested that RF1 specifically interacts with +4 Cs. An estimate for the overall contribution of RF1 to sequence context-dependent variation in translational efficiency was calculated from the range of amber stop codon suppression efficiencies across positional GFP reporter variants. Relative sequence-dependent variation in efficiencies suggested that RF1 interactions are responsible for approximately half of the observed variation.

The amber stop codon suppression efficiencies for 14 different double, triple, quadruple and quintuple tyrosine to amber stop codon GFP mutants were measured in both *E. coli* DH10B and C321.ΔA.exp cells. The overall UAG reassignment efficiencies decrease with increasing numbers of UAG codons in the GFP reporters. In both cell lines there is a clear trend that the measured amber stop codon suppression efficiencies are consistently less than what would be predicted from measured single site efficiencies. The most probable explanation for long range synergistic interaction of suppression sites is that increasing demand for translational components reduces the per site suppression efficiency. Average per site reassignment efficiencies decreased and leveled off as the translation component demand increased in DH10B cells (i.e., in the presence of competing release factor). In C321.ΔA.exp cells without competing release factor, the observed per site suppression efficiency was consistent across the set of evaluated multisite mutants.

## Figures and Tables

**Table 1:** Amber stop codon reassignment efficiency for single amber green fluorescent protein (GFP) reporter variants.

Position of UAG Codon	Nucleotide Sequence Surrounding UAG ^1^	Reassignment Efficiency DH10B	Reassignment Efficiency C321.ΔA.exp	*p*-Value (between Strains)
Tyr 66	CUG ACC *UAG* **G**GC GUC	116.9 ± 2.6%	97.5 ± 3.6%	1.93 × 10^−11^
Tyr 74	UCC CGU *UAG* **C**CG GAC	51.2 ± 1.9%	75.5 ± 4.9%	2.18 × 10^−12^
Tyr 92	GAA GGC *UAG* **G**UA CAG	83.6 ± 3.0%	76.4 ± 3.3%	4.05 × 10^−6^
Tyr 106	GGG ACC *UAG* **A**AA ACC	89.7 ± 2.5%	81.5 ± 4.1%	1.34 × 10^−5^
Tyr 143	CUC GAA *UAG* **A**AC UUC	94.9 ± 2.8%	101.9 ± 5.0%	0.000455
Tyr 151	AAC GUA *UAG* **A**UC ACG	95.9 ± 3.1%	97.8 ± 4.9%	0.243
Tyr 182	GAC CAC *UAG* **C**AG CAG	71.1 ± 2.5%	89.9 ± 5.8%	4.38 × 10^−8^
Tyr 200	AAC CAC *UAG* **C**UG UCC	88.5 ± 4.0%	103.7 ± 5.5%	1.14 × 10^−7^

^1^ The targeted amber stop codon (UAG) is italicized. The sequence of the two codons 5′ and two codons 3′ of the targeted codon are shown. The nucleotide in the +4 position is shown in bold type.

**Table 2:** Predicted and measured UAG reassignment efficiencies for reporter variants containing multiple amber stop codons.

Position of UAG Codons	Predicted Reassignment Efficiency DH10B	Measured Reassignment Efficiency DH10B	Predicted Reassignment Efficiency C321.ΔA.exp	Measured Reassignment Efficiency C321.ΔA.exp
Y74, Y182	36.4 ± 1.8%	28.7 ± 1.2%	67.8 ± 6.2%	52.7 ± 3.2%
Y66, Y143	111.0 ± 4.1%	70.6 ± 3.2%	99.4 ± 6.1%	63.3 ± 4.6%
Y66, Y74	59.8 ± 2.6%	33.7 ± 0.9%	73.6 ± 5.5%	51.6 ± 6.5%
Y74, Y143	48.6 ± 2.3%	58.8 ± 1.4%	76.9 ± 6.3%	60.7 ± 8.1%
Y74, Y200	45.3 ± 2.7%	37.9 ± 1.8%	78.3 ± 6.6%	70.0 ± 7.3%
Y74, Y92	42.8 ± 2.2%	33.9 ± 1.7%	57.7 ± 4.5%	49.3 ± 5.0%
Y143, Y200	84.0 ± 4.5%	68.9 ± 3.7%	105.7 ± 7.6%	76.4 ± 2.1%
Y143, Y182	67.4 ± 3.1%	54.0 ± 2.3%	91.5 ± 7.4%	68.8 ± 1.8%
Y106, Y143	85.2 ± 3.5%	66.6 ± 2.4%	83.0 ± 5.9%	67.2 ± 0.4%
Y92, Y143	79.4 ± 3.7%	57.9 ± 2.2%	77.8 ± 5.1%	57.2 ± 6.3%
Y74, Y92, Y200	37.9 ± 2.6%	26.2 ± 0.9%	59.8 ± 5.6%	56.0 ± 2.8%
Y74, Y182, Y200	32.2 ± 2.2%	24.7 ± 0.8%	70.3 ± 7.4%	66.0 ± 1.5%
Y74, Y92, Y106, Y200	34.0 ± 2.5%	19.8 ± 0.3%	48.7 ± 5.2%	51.3 ± 1.5%
Y74, Y92, Y106, Y182, Y200	24.2 ± 2.0%	13.2 ± 0.3%	43.8 ± 5.5%	40.0 ± 1.5%

**Table 3:** Average measured ***per codon*** reassignment efficiency for UAG containing reporter variants.

Reporter Variant Class	Number of Systems Evaluated	Number of Codons Considered	Average Measured per Site Incorporation Efficiency DH10Bs ^1^	Average Measured per Site Incorporation Efficiency C321.ΔA.exp
1 UAG codon	8	8	86.5%	90.5%
2 UAG codons	10	20	70.6%	78.4%
3 UAG codons	2	6	63.3%	84.7%
4 UAG codons	1	4	66.7%	84.6%
5 UAG codons	1	5	66.7%	83.3%

^1^ The per site reassignment efficiencies were the averages of values determined for genes with equivalent numbers of amber codons. The per site reassignment was calculated as the nth root of the total reassignment efficiency, where n is equal to the number of amber codons in the gene.

**Table 4:** Instantaneous doubling times for amber stop codon suppressing systems.

Cellular Environment	Instantaneous Doubling Time (DH10B)	Instantaneous Doubling Time (C321.ΔA.exp)
**Cells only** (no antibiotic burden)	31.1 ± 1.2 min	40.0 ± 2.7 min
**Inactive tRNA/aaRS and single site GFP reporter** (2 antibiotics, no amber reassignment)	40.9 ± 0.9 min	46.1 ± 4.0 min
**Amber suppressing tRNA/aaRS and single site GFP reporter** (2 antibiotics, with amber reassignment)	54.1 ± 1.8 min	43.9 ± 1.7 min

aaRS: Aminoacyl tRNA synthetase.

**Figure A1:**
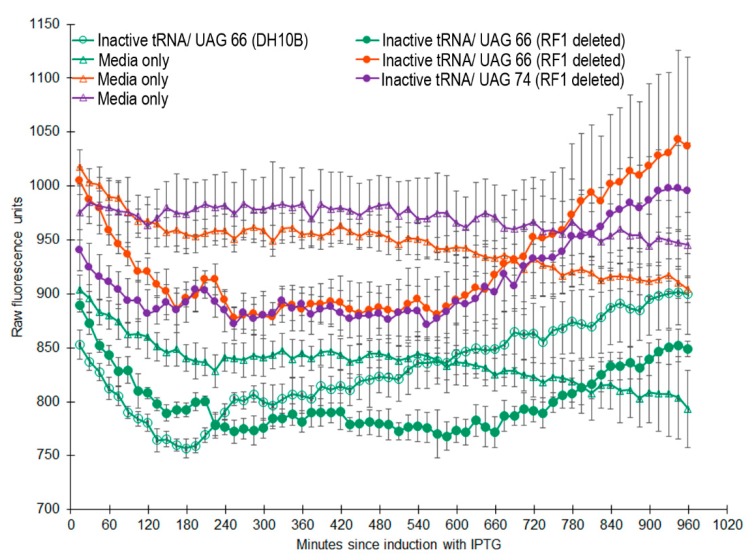
Raw fluorescence versus time profiles of single site UAG GFP reporter variants in the absence of an *M. jannaschii* tRNA capable of suppressing amber codons. Error bars represent the standard deviation across 6 biological replicates of each cell system (12 wells for media only). Data from experiments using two different media preparations are shown and reveal the contribution of media fluorescence to total fluorescence observed in non-reassigning systems.

## Appendix A

**Table A1:** Sequences of mutagenic primers for generating amber stop codon-containing GFP reporter variants.

GFP Position to Be Mutagenized	Primer Sequence (5′ to 3′)
66	CACTCGTCACCACACTCACCTAGGGCGTCCAGTGCTTCTCCCG
74	TCCAGTGCTTCTCCCGTTAGCCGGACCACATGAAACGG
92	GCCATGCCCGAAGGCTAGGTACAGGAACGTACCATCTCCTTC
106	CTTCAAAGACGACGGGACCTAGAAAACCCGTGCCGAAGTC
143	CCTCGGACACAAACTCGAATAGAACTTCAACTCACACAACGTATACATC
151	CAACTTCAACTCACACAACGTATAGATCACGGCAGACAAACAGAAAAAC
182	CCAGCTCGCAGACCACTAGCAGCAGAACACCCCAATCG
200	GTCCTCTTACCAGACAACCACTAGCTGTCCACACAGTCCGTC
237	CGGCATGGACGAACTCTAGAAACACCACCACCACCAC

## Appendix B

Evaluation of raw fluorescence data for systems with little to no expected amber codon reassignment is complicated by the observation that a fraction of the fluorescent components of the media are consumed as cells grow and divide. Various antibiotics also contribute to the background fluorescence of the media; utilizing the corrected fluorescence of non-fluorescent cells grown under different media conditions does not resolve the situation. Minor batch to batch variation in media composition contributes to the majority of differences observed in media fluorescence between experiments. The 0% fluorescent control evaluated in each plate with each batch of media is used to correct the raw fluorescence of systems in which codon reassignment takes place. These impact of these variations on the calculated reassignment efficiency is very low (less than 0.006%, discussed below).

The set of 3 green lines shows the raw fluorescence over time for blank media (open triangles, ∆), an amber GFP reporter co-expressed with inactive orthogonal translation machinery in C321.ΔA.exp (closed circles, ●) and DH10B (open circles, ○). As DH10B cells express RF1 and can terminate translation at UAG codons in the absence of an amber suppressing tRNA, one does not expect near-cognate suppression of the UAG codon by endogenous tRNAs. The raw fluorescence observed in both strains is similar as a function of time, which suggests that suppression of amber codons by near-cognate tRNAs is not significantly contributing to the fluorescence observed in reassignment systems expressing functional amber suppressing tRNAs and UAG GFP reporter variants.

The set of maroon and purple lines shows the raw fluorescence over time for blank media (open triangles, ∆) and either the GFP position 66 (closed maroon circles, ●) or GFP position 74 (closed purple circles, ● UAG variants co-expressed with inactive orthogonal translation machinery in C321.ΔA.exp cells. These two evaluations were performed on consecutive days using the same media, as noted by the similar raw fluorescence traces for blank media. The raw fluorescence versus time profiles for the 2 reporter variants are nearly identical. As noted above, in the case of the position 66 variant, only suppression by tyrosine would result in observed fluorescence, while in the case of the position 74 variant, suppression by any amino acid could lead to fluorescence (assuming that position 74 of GFP is tolerant to mutation). For these reasons, despite the absence of mass analysis, we do not suspect that suppression of the amber stop codons by near-cognate tRNA in the C321.ΔA.exp strain is contributing to our calculations of amber suppression efficiency.

Further, estimates of the relative contribution of possible suppression by near-cognate endogenous tRNAs would comprise very little of the overall reassignment efficiency as a percent of the 100% fluorescent wild type GFP. Raw fluorescence for the wild type GFP in these experiments reaches approximately 33,000 raw fluorescence units. 200 units of raw fluorescence (an estimate larger than would be expected by the representative raw profiles shown), would adjust the calculated reassignment efficiency by 0.006%, a value below the limit of detection of our in cell assay, which we have calculated to be 0.015% (Discussed in detail in [44]).
